# Neurosymptomatology

**Published:** 1891-09

**Authors:** 


### NEUROSYMPTOMATOLOGY.

ALBUMINURIA IN EPILEPSY.—The American Lancet, in an editorial comment, says:

"In our own experience, albuminuria is exceedingly rare in epilepsy, and from some hundreds of examinations of such urine in upwards of fifty cases we have failed to find albumen a single time in uncomplicated epilepsy. Once albumen was found in large quantity, and the autopsy revealed advanced tubercular nephritis. The examinations were made with urine obtained before, during and after paroxysms, as well as in the interval between the attacks. These results would emphasize the assertion that albuminuria is very infrequent in such cases, and that renal disease is mainly responsible for its occurrence. Kidney disease should be suspected when albumen appears more than transiently in the urine.

"It must not be forgotten, that in doubtful cases, uræmic and epileptic convulsions may co-exist, and here, if a history of the case is wanting, one or the other conditions may remain unsuspected, no differentiated point existing in the seizures themselves. This combination we believe to be a very rare one, but we have seen a case in which both factors—essential epilepsy and uræmic toxhæmia—undoubtedly each contributed a share in the product of the oft-repeated epileptic status. Fortunately such a complication is rare, and it would seem that epileptics as a class suffer very little comparatively from diseases of the kidney producing albuminuria.

"In conclusion we would again direct attention to this interesting question, and hope that those so fortunately placed as to observe large numbers of this unfortunate class will contribute further statistics of the frequency of albuminuria in simple epilepsy without kidney disease."

[We have examined the urine of hundreds of epileptics and never found albumen persistently present in any uncomplicated case of epilepsy, and in not one per cent. have we seen even slight and transient traces of albumen in the urine of epileptics. In this disease the urine is singularly free of this sign of albuminuria.—Ed.]—*Alienist and Nerologitus.*

